# Physical Activity in Pulmonary Arterial Hypertension during Pandemic COVID-19 and the Potential Impact of Mental Factors

**DOI:** 10.3390/ijerph19148343

**Published:** 2022-07-08

**Authors:** Maria Wieteska-Miłek, Sebastian Szmit, Michał Florczyk, Anna Witowicz, Marcin Kurzyna

**Affiliations:** Centre of Postgraduate Medical Education, European Health Center, Department of Pulmonary Circulation, Thromboembolic Diseases and Cardiology, 05-400 Otwock, Poland; sszmit@cmkp.edu.pl (S.S.); michal_florczyk@wp.pl (M.F.); anna.witowicz@ecz-otwock.pl (A.W.); mkurzyna@cmkp.edu.pl (M.K.)

**Keywords:** physical activity, pulmonary arterial hypertension, COVID-19 pandemic, fear of COVID-19, HADS scale

## Abstract

One of the non-pharmacological recommendations for stable patients with pulmonary arterial hypertension (PAH) is to increase physical activity. The study aimed to analyze the degree of physical activity of PAH patients and check if mental factors may have a potential negative impact during the COVID-19 pandemic. Forty patients with stable PAH were included in the study. Physical activity was assessed by pedometer (Omron HJ-321-E) for four weeks. At baseline, in addition to the 6 min walk test (6MWT) and functional assessment, patients completed the quality-of-life questionnaire SF-36, fear of COVID-19 scale, and hospital anxiety and depression scale (HADS). The mean age of the study group was 45.5 years, 80% were women, and 62.5% had idiopathic/heritable PAH. Low physical activity defined as <5000 steps/day had 19 (47.5%), and moderate/high physical activity (≥5000 steps/day) had 21 (52.5%) patients. Patients with low physical activity less frequently worked compared with the moderate–high-activity sub-group, 42% vs. 81%, *p* = 0.03, and had the shorter distance in 6-6MWT, *p* = 0.03. There was no significant correlation between steps/day and different mental factors. Almost half of the study group had low activity during the pandemic. Mental factors did not impact physical activity in PAH patients during the pandemic.

## 1. Introduction

Pulmonary arterial hypertension (PAH) is a rare disease characterized by elevated mean pulmonary arterial pressure, elevated pulmonary vascular resistance, and normal pulmonary artery wedge pressure in right heart catheterization, without other causes of pre-capillary pulmonary hypertension [1]. If left untreated, the disease leads to progressive right ventricular failure, the development of the low-output syndrome, and death [1]. Exercise intolerance, fatigue, and dyspnea are the symptoms of decreased quality of life and worse prognosis [1]. Some studies show that PAH patients reduce physical activity [2,3].

Stable PAH patients should be encouraged to be active; they can significantly benefit from supervised exercise rehabilitation [4]. In randomized controlled trials, PAH patients who reached higher levels of physical activity improved distance in 6MWT, cardiorespiratory function, and quality of life compared with untrained controls [5,6].

Guidelines recommend that PAH patients be active, but not the all mechanisms that affect physical activity in PAH patients are well known. One study suggested that the presence of depression symptoms negatively impacted physical activity in PAH patients [7]. The COVID-19 pandemic increased the risk of isolation, anxiety, and depression, potentially negatively impacting physical activity and mental health worldwide. The most vulnerable groups are patients with life-threatening diseases, including PAH and CTEPH.

Our study aimed to measure the degree of physical activity of PAH patients, fear of COVID-19, anxiety, and depression and assess if mental factors had a potentially negative impact on physical activity during the COVID-19 pandemic in PAH patients.

## 2. Materials and Methods

### 2.1. Study Group

The prospective cross-sectional study was performed in the pulmonary hypertension center in Poland during the third and fourth waves of the COVID-19 pandemic. Consecutive PAH patients who received stable targeted therapy for at least three months while visiting our center were considered for inclusion in the study. All patients were 18 years old or older and had a diagnosis of PAH confirmed by right heart catheterization and additional necessary compulsory tests according to the current guidelines [1]. Exclusion criteria were cognitive impairment to completing the questionnaire, mobility impairment due to musculoskeletal or neurological disease, and lack of consent to participate in the study. Data of mean age, range of age variation, percentage of women and men, type of PAH, disease characteristics, concomitant diseases, specific PAH treatment, and working status were collected at baseline. The study protocol was approved by the Bioethics Committee of the Centre of Postgraduate Medical Education under the Declaration of Helsinki (number KBE 22/2021, date of approval: 14 April 2021). Written informed consent was obtained from all participants.

### 2.2. Methods

At the baseline visit, all the participants completed a six-minute walk test (6MWT) according to the American Thoracic guidelines [8], blood sampling for NT-proB-type Natriuretic Peptide (NT-proBNP) level, and half of them had transthoracic echocardiography. These non-invasive tests are routinely used according to the current guidelines for the prognostic assessment of PAH patients [1]. In 6MWT, the distance, oxygen saturation before and after the test, and Borg dyspnea score before and after the test were measured. In transthoracic echocardiography, systolic pulmonary arterial pressure, right atrial area, tricuspid annular plane systolic excursion, and presence of fluid in the pericardium were collected for further analysis.

All the patients were asked to fill in four questionnaires in the Polish version: Quality-of-life questionnaire (SF-36 v.2), the fear of COVID-19 scale (FCV-19S), the hospital anxiety and depression scale (HADS), and the acceptance of illness scale (AIS). To assess the quality of life, a license number QM 057109 was obtained for using the 36-item Short Form (SF-36, v.2). The SF-36 consists of 8 scales: physical functioning (PF), role-physical (RP), bodily pain (BP), general health (GH), vitality (VT), social functioning (SF), role-emotional (RE) and mental health (MH). The first four summarized components were used for evaluating the physical component summary (PCS): PCS = PF + RP + BP + GH. The summary of the following four components was used to evaluate the mental component summary (MCS): MCS = VT + SF + RE + MH [9]. The scored items for each variable were coded and transformed on a scale from 0 (worst possible health state) to 100 (best possible health state). The rule is that the higher the index, the more self-esteem of the respondent, in terms of what positively contains the accepted concepts of the quality of life. The fear of COVID-19 scale is a psychometric tool for measuring the anxiety due to the COVID-19 pandemic. It consists of seven items. Answers were given on a scale ranging from 1 (strongly disagree) to 5 (strongly agree). Each patient chose a point from 1 to 5 for each statement and could obtain a total score of 7 to 35 points [10]. The higher the points, the higher the fear of COVID-19 [10]. The FCV-19S has been translated into the Polish language and validated for the Polish population [11,12]. The hospital anxiety and depression scale reflect the general level of anxiety and depression. It consists of 16 items divided into anxiety (seven questions), depression (seven questions), and anger (two questions). Each item has four possible answers, and 0 to 21 points can be obtained for each sub-scale of anxiety or depression [13]. The exact cut-off value of eight or more in the HADS anxiety part (HADS-A) or HADS depression part (HADS-D) can be used to determine whether patients are anxious or depressed [14]. The HADS was translated into the Polish language and validated for the Polish population [14]. The acceptance of illness scale is the tool used to assess the acceptance of the limitations caused by the illness. The scale contains eight statements followed by a 5-choice rating. Answers were given on a scale ranging from 1 (strongly agree) to 5 (strongly disagree), ranging from eight to 40 points. The higher the points, the higher the acceptance of the disease. A score of ≤18 denotes a lack of illness acceptance, and ≥30 denotes a high level of illness acceptance. The AIS was translated into the Polish language and validated for the Polish population [15,16].

Each participant received a pedometer (Omron HJ-321-E)-Omron walking style. It is a small device: 75.0 mm × 31 mm × 8.0 mm and light, weighing 20 g [17]. This activity monitor was selected because it is widely available, cheap, and straightforward. Other studies checked its accuracy in measuring physical activity [18,19]. All patients were instructed to engage in regular physical activity for four weeks and to wear the pedometer constantly except when showering or sleeping.

As the pedometer has a 7-day memory, patients were asked to write down their step results in a diary. According to the manufacturer’s instructions, the pedometer could be worn attached to belt or pants, in a pocket of clothing, or in a bag [17]. All patients were instructed on how to wear it correctly. Patients measured steps for four weeks; after that, a teleconsultation with the doctor was performed to discuss obtained results. The number of 5000 steps per day was established to distinguish between low physical activity and moderate–high-activity subgroups. The number of steps around this value was a predictor of hospitalization in PAH patients [20] and a predictor of death in chronic heart failure patients [21]. Medical records were reviewed to obtain information about the demographic characteristics of the patients, their clinical conditions, and the treatment they had received.

### 2.3. Statistical Analysis

Statistical analysis was performed using Statistical software (by TIBCO Software Inc., license acquired from local distributor StatSoft Polska, Krakow, Poland), version 13.3. Data distribution was tested using the Kolmogorov–Smirnov test. Categorical variables were presented as numbers and percentages; continuous variables were presented as medians and interquartile ranges or means and standard deviations. Chi-square, Fisher’s exact-test, paired *t*-test, or the Mann–Whitney U test were used as appropriate for group comparisons. Spearman correlation test was used to assess the relationship between parameters. The relationship differences were considered significant for *p* ≤ 0.05.

## 3. Results

### 3.1. Study Group

A total of 60 consecutive PAH patients were screened as potential study participants. Two of them were incapable of providing informed consent due to mental health problems, three had mobility problems, and fourteen had changes in targeted therapy less than three months before the study. Forty-one patients with PAH were enrolled in the study; one of them withdrew his consent and did not complete the study. Forty patients’ data were included in the final analysis. Compliance in measuring steps in the study group was high, from 89% to 100%.

Most of the patients were female, 32 (80%). The patients’ mean age was 45.5 years (ranging from 24 to 64). In the study group, 22 (55%) patients suffered from IPAH, and nine (22.5%) patients had PAH due to connective tissue disease. The clinical characteristics of the study group are presented in Table 1.

### 3.2. Physical Activity in Study Group

**Figure 1:**
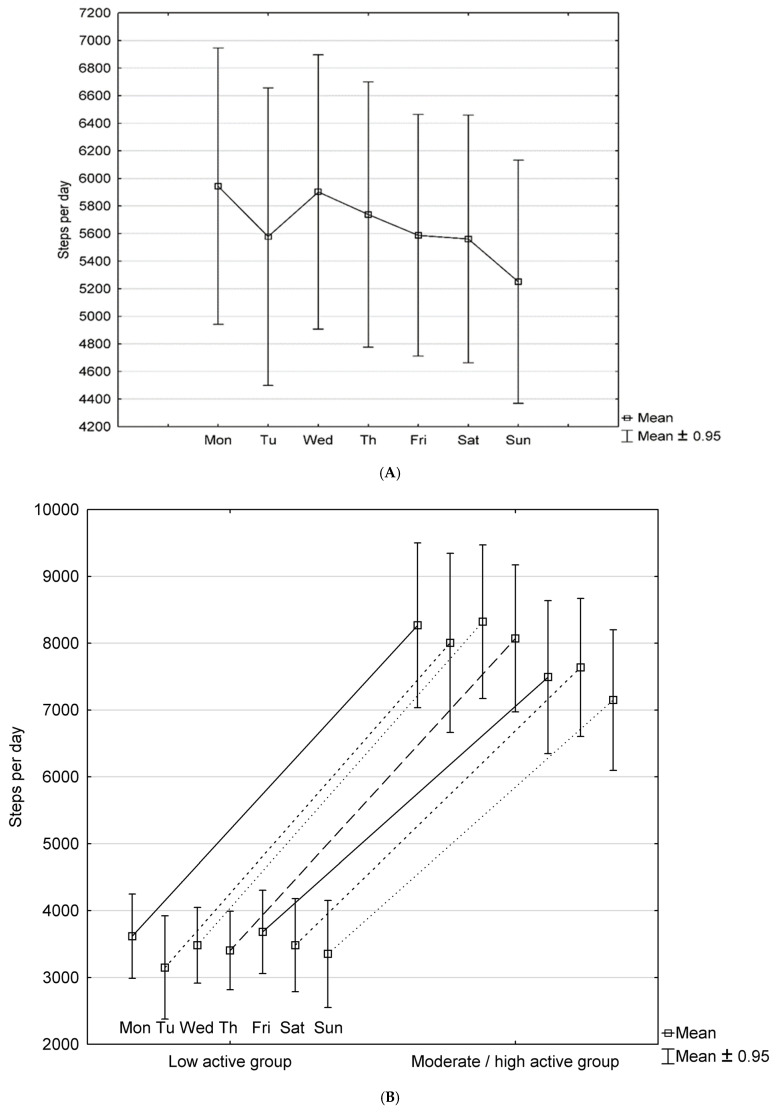
(**A**) Daily steps count for the whole group of PAH patients—variability in mean steps/day across the week. (**B**) Daily steps count for the low-activity and moderate–high-activity subgroups of PAH patients—variability in mean steps/day across the week.

The mean daily steps count was 5654 ± 2687. The mean number of steps/day for weekdays was 5775 ± 2826 and for weekends 5391 ± 2528. We divided the study groups into two sub-groups: low-activity PAH patients defined as <5000 steps per day and moderate–high-activity PAH patients with physical activity ≥5000 steps per day. The results for individual days of the week for the whole group, low-activity and moderate–high-activity subgroups are presented in Figure 1.

Low physical activity was observed in 19 (47.5%) of PAH patients, moderate physical activity (5000–7499 steps per day) in 10 (25%) of patients, and high physical activity (≥7500 steps per day) in 11 (27.5%) of PAH patients.

Mean steps per day in the low-activity group was 3393 ± 1039, while in the moderate-high active group was 7701 ± 1039. Patients with low-activity group differ from the moderate-high active group not only by daily steps count but, in terms of distance in 6MWT (502.7 ± 65.5 vs. 549.1 ± 65.4, *p* = 0.03) and percentage of working patients [8 (42%) vs. 17 (81%), *p* = 0.03]. There was no difference in the type of PAH, the WHO functional class, other functional parameters, NTproBNP level, the proportion of parenteral prostacyclin, combined therapy or home oxygen treatment between both groups. Baseline characteristics comparison between low-activity and moderate–high-activity sub-group are presented in Table 1 and Table 2.

### 3.3. Fear of COVID-19, Anxiety and Depression and Quality of Life in Study Group

Patients with PAH in the study groups had impaired quality of life, both physical and mental components. Nine (22.5%) of PAH patients had a high level of anxiety, and four (10%) of patients had depression. Five patients with anxiety and three with depression were in the moderate–high-activity group. There was no significant difference in the quality of life, fear of COVID-19, hospital anxiety and depression, or acceptance of the illness between the low- and high–moderate-activity groups. The results are presented in Table 3.

There was a correlation of steps/day with 6MWT distance but not with psychological parameters (Table 4).

## 4. Discussion

The COVID-19 pandemic impacted the lives of all people. The risk of death among PAH and CTEPH patients due to the COVID-19 disease is much higher than the general population and amounts to 16–21% [22,23]. The COVID-19 pandemic is associated with social isolation and has worsened the mental state of healthy people [24] and people with life-threatening diseases [25,26].

To the best of our knowledge, this is the first prospective study to assess physical activity in PAH patients during the COVID-19 pandemic. We found that 47.5% of prevalent stable PAH patients had low physical activity, defined as a daily step count <5000 steps per day. It is less frequent than in incident PAH/CTEPH patients, where 64% of them had a low physical activity before the pandemic [2]. Our study was performed during the third and fourth waves of the COVID-19 pandemic. At that time, there were no restrictions on staying outside the home, but it was necessary to wear masks indoors; there were restrictions on the maximum number of people in shops and churches and a ban on mass events. Although it was not the time of the most significant restrictions, physical activity among the studied group of PAH patients was low and cannot be justified by anxiety or depression related to the pandemic.

There is no current clear definition of low physical activity for PAH patients. The Tudor-Locke et al. defined the sedentary lifestyle index as <5000 steps per day in the general population [27]. Physical activity less than 5000 steps per day is associated with cardiometabolic risk factors and unfavorable body composition in healthy people [27]. A representative sample of U.S. adults showed that a more significant number of steps per day was significantly associated with lower all-cause mortality [28]. Compared with individuals who took 4000 steps per day, taking 8000 steps per day was associated with lower all-cause mortality (a hazard ratio of 0.49). A meta-analysis of 15 studies was observed progressively decreasing the risk of mortality among adults aged 60 years and older with an increasing number of steps per day until 6000–8000 steps per day and among adults younger than 60 years until 8000–10,000 steps per day [29]. Results in heart failure patients are different than in the general population. Prospective observation of outpatients with left heart failure showed physical activity of ≤4889 steps/day to be a strong and independent predictor of mortality in multivariate analysis [21]. In another study, Marvin-Peek et al. observed that baseline step count was associated with the risk of future hospitalization in PAH patients during two years of follow-up. The median step count for hospitalized PAH patients was 3899 steps per day compared with 5367 steps per day in those who did not need hospitalization [20]. We chose a cut-off value of 5000 steps per day for the present study because we hypothesized that the number of steps around 5000 per day might predict hospitalization and death for PAH patients. Further studies are needed to prove this hypothesis. 

Our cohort of PAH patients’ mean steps per day were 5654 ± 2687. It is much higher than in a previously published studies before the pandemic, where in prevalent PAH/CTEPH patients, the mean steps were (4976.7 steps/day) [30], 4280 ± 2351 steps per day [7], or 5041 ± 3357 steps per day [31]. The low-activity subgroup differed from the more active subgroup in the 6 min walk test distance (shorter in the low-activity group) and in the percentage of working people (lower in the low-activity group). Interestingly, neither subgroup differed in demographic parameters, age, the WHO functional class, NTproBNP level, functional echocardiographic parameters, PAH treatment method, including parenteral prostacyclin, comorbidities, mental status, or quality of life.

In our study groups, the mean walking distance in 6MWT was 527.1 ± 68.7 m. It is much higher than that in the Polish registry of PAH patients, where it was 375 ± 142 m [32]. Our study group was younger, had a better WHO functional class, and had a lower NTproBNP level. Moreover, there was no incidence of patients in our study groups, and all patients had stable disease at least three months before the study. We observed that the working PAH patients had higher daily steps count than retirees or pensioners. Physical activity in PAH patients is higher on weekdays than on weekends. Similar relationships were observed in the general population and young adults [33,34].

Fear is an adaptive defense mechanism fundamental and instrumental to survival. The FCV-19S is a psychometric tool created during the beginning of the SARS-CoV-2 pandemic, to assess emotional response to the pandemic, reflecting depression, anxiety, stress, and mental well-being. Median FCV-19S in our study group was 15.0 (12.0–22.5), higher than the general Polish population [12]. Despite the fear of COVID-19, PAH patients were not less active than before the pandemic, even though they needed to maintain social distancing. There is no significant difference in FCV-19S between low-activity and moderate–high-activity patients. Moreover, our study showed that FCV-19S did not significantly affect the number of steps per day, and there is no correlation between FCV-19S and 6MWT distance or the number of steps per day in PAH patients. In the literature, presently only one pilot study performed in Arabic general population, suggesting that FCV-19S and general distress negatively correlate with physical activity, but physical activity was measured by questionnaire, not by steps [35].

The level of anxiety and depression in PAH patients is higher than in the general population. During the SARS-CoV-2 pandemic, around 32% of 223 PAH/CTEPH patients had anxiety, and 21% had depression measured by the HADS scale [25]. WHO functional class 3 or 4 and the history of COVID-19 were found as the primary determinants of depression on the HADS scale in PAH/CTEPH patients during the pandemic. Fear of COVID-19 and history of COVID-19 disease were found as the primary determinants of anxiety on the HADS scale in PAH/CTEPH patients [25]. In another paper by Park et al., among 152 PAH patients, 34% had anxiety, and 23% had depression on the HADS scale during the pandemic. Interestingly, the prevalence of mental disorders did not change after 232 days of observation, suggesting that the pandemic had little effect on anxiety and depression in patients with PAH [26]. In our present study, the prevalence of anxiety was 21%, and the prevalence of depression 10% on the HADS scale. Our results may be because our study group was younger, had a better WHO functional class, had a less frequent history of COVID-19 disease, and had a lower fear of COVID-19 compared with those in previous studies [25,26].

Twenty-five percent of the study participants were unvaccinated. There was no significant difference in the HADS, FCV-19, SF-36 scores, daily steps, and 6MWT distance between the vaccinated and unvaccinated patients. Another study investigating the tolerance and reason for refusing COVID-19 vaccination showed that only advanced age ≥ 60 years was a determinant of willingness to vaccinate against COVID-19 in PAH/CTEPH. Mental factors had no impact on making decisions [36].

We observed a significant correlation between daily steps count and 6MWT distance compared with other studies [2,7,31,37]. There was no correlation between mental parameters, anxiety and depression, and daily step counts and distance in 6MWT. This is different from Nakazato et al., who found a significant negative correlation between HADS-D and the number of steps per day [7]. Our study population was larger, had a longer 6MWT distance at baseline, and a higher mean daily step count, and fewer of our patients had symptoms suggestive of depression on the HADS scale. It is unclear if depression affects exercise capacity and physical activity or vice versa. Depression may impact physical activity in PAH patients, but this hypothesis needs further studies. In other diseases, for example, people at high risk of coronary vascular disease with depressive symptoms were less active than patients without depression [38].

We found, as in the paper by Somaini et al., negative correlation between HADS score and quality of life [39]. Similar to other studies, a correlation between 6MWT distance and HADS scale was not observed [2,7,39]. The quality-of-life questionnaire makes it possible to assess the physical and mental components of the patient’s functioning from the patient’s point of view. When patients have depression, their emotional quality of life deteriorates; this is important from a practical point of view to not associate a reduced quality of life in depressive PAH patients with poor PAH control but effectively treatable comorbidity.

Our study has some limitations. It is a single-center study with a relatively small study group. The time from completion of the questionnaire and time to start measuring steps from the first patient to the last patient was long, taking eight months. Patients did not have measured physical activity before the pandemic, so it is impossible to investigate how it changed due to the pandemic, but only to determine what it was. We conducted our study during the third and fourth waves of the COVID-19 pandemic. The level of anxiety or depression might have varied at different time points in the pandemic, but the paper shows that the frequency of anxiety and depression in PAH patients in the pandemic measured by HADS scale is unchanged.

## 5. Conclusions

This study suggests that around half of the stable PAH patients had low physical activity during the COVID-19 pandemic and were less active during the weekends than on weekdays. Mental factors, such as fear of COVID-19, anxiety, and depression, had no impact on physical activity in PAH patients. The obtained results suggest a strong need for interventions increasing physical activity, especially in non-working patients and on weekends.

## Figures and Tables

**Table 1:** Clinical characteristics of the study group.

Clinical Parameters	Total PAH Study Group n (%) or Mean (SD)	Low-Activity PAH Subgroup n (%) or Mean (SD)	Moderate–High-Activity PAH Subgroup n (%) or Mean (SD)	Low-Activity vs. Moderate–High-Activity Subgroup *p*-Value
Number of patients	40 (100%)	19 (47.5%)	21 (52.5%)	
Age, years	45.5 (24.7–64.4)	44.3 (27.8–64.4)	46.5 (24.7–61.3)	0.5
Sex, female	32 (80%)	13 (68%)	19 (90%)	0.2
Duration of disease, years	7.6 (6.7)	7.6 (6.9)	7.7 (6.7)	0.96
Idiopathic PAH	22 (55%)	10 (53%)	12 (57%)	1.0
Heritable PAH	3 (7.5%)	0	3 (14%)	
PAH associated with CHD	5 (12.5%)	3 (16%)	2 (9.5%)	
PAH associated with CTD	9 (22.5%)	6 (32%)	3 (14%)	
PAH porto-pulmonary	1 (2.5%)	0	1 (5%)	
PDE5/sCG	35 (87.5%)	19 (100%)	16 (76%)	0.2
ERA	30 (75%)	13 (68%)	17 (81%)	0.5
Prostacyclin/IP receptor agonist	20 (50%)	13 (68%)	7 (33%)	0.06
One PAH drug	11 (27.5%)	4 (21%)	7 (33%)	0.1
Two PAH drugs	13 (32.5%)	4 (21%)	9 (43%)	
Three PAH drugs	16 (40%)	11 (58%)	5 (24%)	
WHO functional class	2.1 (0.42)	2.2 (0.37)	2.0 (0.45)	0.4
1	2 (5%)	0	2 (9.5%)	
2	33 (82.5%)	16 (84%)	17 (81%)	
3	5 (12.5%)	4 (16%)	2 (9.5%)	
History of COVID-19 disease	4 (10%)	2 (10%)	2 (9.5%)	0.96
Vaccination against COVID-19	30 (75%)	15 (79%)	15 (71%)	0.7
Working	25 (62.5%)	8 (42%)	17 (81%)	0.03 *
Concomitant disease #	18 (45%)	7 (37%)	11 (52%)	0.4
Obesity, BMI ≥ 30 kg/m^2^	8 (20%)	2 (10%)	6 (29%)	0.3

BMI—body mass index; PAH—pulmonary arterial hypertension, PAH-CHD—pulmonary arterial hypertension related to congenital heart disease, IPAH—idiopathic pulmonary arterial hypertension, PAH-CTD—pulmonary arterial hypertension associated with connective tissue disease, PAH—porto-pulmonary-pulmonary arterial hypertension associated with portal hypertension, ERA—endothelin receptor agonists PDE5—phosphodiesterase 5-inhibitors, sGC—soluble guanylate cycle stimulator; IP—prostacyclin receptor; WHO—World Health Organization; # arterial hypertension/chronic obstructive pulmonary disease/coronary artery disease/neo-plasm/diabetes or obesity; * *p* < 0.05.

**Table 2:** Baseline characteristics of the study group.

Functional Parameters	Total Study Group n (%) or Mean (SD) or Median (IQR)	Low-Activity Subgroup n (%) or Mean (SD) or Median (IQR)	Moderate/ High-Active Subgroup n (%) or Mean (SD) or Median (IQR)	Low-Activity Subgroup vs. Moderate–High-Activity Subgroup *p*-Value
WHO functional class	2.1 ± 0.42	2.15 ± 0.37	2.0 ± 0.45	0.4
NT-proBNP, ng/L	150.5 (91–351)	211.5 (93–452)	110 (82–255)	0.07
6MWT distance, m	527.1 ± 68.7	502.7 ± 65.5	549.1 ± 65.4	0.03 *
Oxygen saturation before 6MWT, %	95.5 ± 3.6	94.8 ± 4.2	96.1 ± 2.3	0.3
Oxygen saturation a after 6MWT, %	91.1 ± 7.3	89.7 ± 8.7	92.2 ± 5.7	0.5
Borg before 6MWT, points	0.2 ± 0.4	0.2 ± 0.5	0.2 ± 0.4	0.7
Borg at the end of 6MWT, points	2.4 ± 1.2	2.2 ± 1.3	2.5 ± 1.2	0.7
sPAP, mmHg	n = 20; 62.8	n = 11; 62.2	n = 9; 63.3	0.6
TAPSE, mm	n = 23; 22.9	n = 13; 21.8	n = 10; 24.4	0.3
RAA, cm^2^	n = 23; 23.0	n = 13; 23.5	n = 10; 22.5	0.8
Presence of fluid in pericardium, yes/no	n = 23; 4 (10%)	n = 13; 3 (16%)	n = 10; 1 (5%)	0.6

6MWT—six-minute walk test; NTproBNP—NT-proB-type Natriuretic Peptide; sPAP—systolic pulmonary arterial pressure; TAPSE—tricuspid annular plane systolic excursion, RAA—right atrium area; WHO—World Health Organization; IQR—interquartile range; * *p* < 0.05.

**Table 3:** Quality of life, fear of COVID-19, hospital anxiety and depression, acceptance of the illness among low-activity and moderate–high-activity PAH patients.

Variables	Total PAH Study Group Median (IQR) n = 40	Low-Activity PAH Group Median (IQR) n = 19	Moderate–High-Activity PAH Group Median (IQR) n = 21	Low-Activity vs. Moderate/ High Active PAH Group *p*-Value
PCS-SF-36, points	44.9 (38.1–49.5)	45.2 (37.7–49.4)	54.7 (38.3–51.6)	0.6
MCS-SF-36, points	49.8 (44.6–57.2)	49.9 (44.3–59.1)	48.6 (47.2–55.9)	0.7
FCV-19S, points	15.0 (12.0–22.5)	14 (12–20)	17 (12–23)	0.4
HADS-A, points	5.0 (2.5–6.5)	5 (2–6)	5 (3–7)	0.8
HADS-D, points	2 (1–4)	2 (1–5)	2 (1–3)	0.8
HADS-A ≥ 8	9 (22.5%)	4 (21%)	5 (24%)	0.9
HADS-D ≥ 8	4 (10%)	1 (5%)	3 (14%)	0.6
AIS, points	29 (23.5–33.5)	28 (25–33)	30 (23–34)	0.5

AIS—acceptance of illness scale, FCV—19S-fear of COVID-19 scale, HADS—hospital anxiety and depression scale, HADS-A—anxiety part, HADS-D—depression part; MCS—mental summation score; PCS—physical summation score; PAH—pulmonary arterial hypertension, SF-36—36-item short-form survey; IQR—interquartile range.

**Table 4:** Correlation between physical activity, 6 min walk test distance and quality of life, fear of COVID-19, hospital anxiety and depression, acceptance of the illness in PAH patients (n = 40).

Variables	Steps/Day	*p*-Value	6MWT Distance, m	*p*-Value
6MWT distance, m	0.41	<0.01 *	-	-
PCS-SF36, points	0.08	0.6	0.27	0.09
MCS-SF-36, points	−0.17	0.3	−0.007	0.9
FCV-19S, points	0.02	0.9	−0.02	0.8
HADS-A, points	0.19	0.2	0.005	0.9
HADS-D, points	0.17	0.3	−0.18	0.3
AIS, points	0.03	0.9	0.08	0.6

6MWT—six-minute walk test, PCS-SF-36—physical summation score SF-36, MCS-SF-36—mental summation score SF-36, AIS—acceptance of illness scale, FCV—19S-fear of COVID-19 scale, HADS—hospital anxiety and depression scale, HADS-A—anxiety part, HADS-D—depression part; PAH—pulmonary arterial hypertension; SF-36—36-item short-form survey; * *p* < 0.05.

## Data Availability

Data sharing does not apply to this article.
